# Sonographic abnormalities in pregnancies conceived following IVF with and without preimplantation genetic testing for aneuploidy (PGT-A)

**DOI:** 10.1007/s10815-021-02069-5

**Published:** 2021-02-03

**Authors:** Carrie K. Riestenberg, Thalia Mok, Jessica R. Ong, Lawrence D. Platt, Christina S. Han, Molly M. Quinn

**Affiliations:** 1 Division of Reproductive Endocrinology and Infertility, Department of Obstetrics and Gynecology, University of California, Los Angeles, 10833 Le Conte Avenue 27-139 CHS, Los Angeles, CA 90095-1740 USA; 2 Division of Maternal-Fetal Medicine, Department of Obstetrics and Gynecology, University of California, Los Angeles, 10833 Le Conte Avenue 27-139 CHS, Los Angeles, CA 90095-1740 USA; 3 Center for Fetal Medicine & Women’s Ultrasound, 6310 San Vicente Blvd, Suite 520, Los Angeles, CA 90048 USA

**Keywords:** IVF, Preimplantation genetic testing (PGT-A), Fetal anomalies

## Abstract

**Purpose:**

To report the rate of fetal anomalies detected on anatomy ultrasound in pregnant patients who underwent IVF with preimplantation genetic testing for aneuploidy (PGT-A) compared to patients who conceived following IVF with unscreened embryos and age-matched patients with natural conceptions.

**Methods:**

Retrospective cohort study at a single maternal-fetal medicine practice. Patients with singleton pregnancies who had a mid-trimester anatomy ultrasound between January 2017 and December 2018 were screened for inclusion. A total of 712 patients who conceived after IVF with or without PGT-A were age-matched with natural conception controls. The primary outcome was the rate of fetal and placental anomalies detected on mid-trimester anatomical survey. Secondary outcomes included the rates of abnormal nuchal translucency (NT), second trimester serum analytes, non-invasive prenatal testing (NIPT), and invasive diagnostic testing.

**Result(s):**

There were no differences in the rate of fetal anomalies in patients who underwent IVF with PGT-A compared to patients who conceived following IVF with unscreened embryos and age-matched patients with natural conceptions. Rate of abnormal NT, high-risk NIPT, and abnormal invasive diagnostic testing were also similar. Patients who conceived after IVF with or without PGT-A had higher rates of abnormal placental ultrasound findings and abnormal second trimester serum analytes compared to natural conception controls.

**Conclusion:**

The use of PGT-A was not associated with a difference in risk of fetal anomaly detection on a mid-trimester anatomical survey. The results of this study highlight the importance of improved patient counseling regarding the limitations of PGT-A, and of providing standard prenatal care for pregnancies conceived through ART, regardless of whether PGT-A was performed.

**Supplementary Information:**

The online version contains supplementary material available at 10.1007/s10815-021-02069-5.

## Introduction

Preimplantation genetic testing for aneuploidy (PGT-A) has been increasingly adopted by in vitro fertilization (IVF) clinics across the USA, with reported use in over 40% of autologous IVF cycles in 2018 [1]. Providers have turned to PGT-A due to the potential for improved pregnancy and live birth rates on a per-cycle basis [2–5]. However, a recent multicenter randomized control trial found no benefit of PGT-A compared to embryo selection by morphology alone [6].

Ethical concerns pertaining to the rapid uptake of the PGT-A technology include safety, cost, and a misunderstanding of the predictive value of PGT-A results. A survey of 300 patients undergoing IVF at a high-volume academic IVF center found that patients often hold misconceptions about the utility of PGT-A, with the majority of patients indicating that their primary reason for pursuing PGT-A was to increase the chance of having a “healthy baby,” and nearly one-fifth of patients indicating that it was to reduce the risk of congenital anomalies or “birth defects” [7].

Abnormalities of fetal anatomy detected on prenatal ultrasound are reported in approximately 3–4% of pregnancies [8]. It is estimated that nearly one quarter of congenital anomalies are secondary to a genetic etiology, and 5–10% result from identifiable environmental or maternal exposures. However, an etiology remains unidentifiable in two-thirds of cases [9, 10]. With a modern ultrasound technology, sensitivity of antenatal ultrasound for the detection of fetal anomalies has significantly improved, with detection rates of approximately 93% for cleft lip and palate, 75–83% for abdominal wall defects, and 53% for selected heart defects [11–13]. Mid-trimester ultrasound is a critical point at which the fetal anatomy is evaluated in detail. Abnormalities detected on mid-trimester ultrasound influence the management of the remainder of the pregnancy and have been found to be significantly associated with the likelihood of pregnancy termination [14].

Few studies have investigated the rate of congenital anomalies in pregnancies resulting from IVF with PGT-A. Zhang et al. found no overall difference in the rate of congenital anomalies between patients who underwent IVF with or without PGT-A followed by frozen or fresh embryo transfer (ET) [15]. Importantly, this study excluded miscarriages and pregnancy terminations for fetal anomalies and will therefore inherently underestimate the prevalence of anomalies in both groups.

The goal of the present study was to report the rates and specific types of fetal and placental anomalies detected on mid-trimester anatomy ultrasound in patients who underwent IVF with PGT-A compared to patients who conceived following IVF with unscreened embryos and age-matched patients with natural conceptions.

## Materials and methods

### Patients

This was a retrospective cohort study at a single high-volume metropolitan maternal-fetal medicine referral center. All patients with singleton pregnancies who had a mid-trimester anatomy ultrasound between January 1, 2017, and December 31, 2018, were screened for inclusion. The electronic medical record was queried to identify patients with singleton pregnancies conceived with IVF with and without PGT-A during the study period. Multifetal gestations and patients without a mid-trimester anatomy ultrasound were excluded. All anatomical surveys at the center were performed by board-certified maternal-fetal medicine physicians using standardized protocols. One-to-one matching with naturally conceived pregnancies during the same period was subsequently performed based on patient age at the time of anatomy ultrasound. For patients who conceived with IVF using donor egg, the age of the egg donor at the time of retrieval was used for age matching.

The charts were systematically reviewed and the data extracted by three members of the research team (CR, TM, JRO) using a shared protocol. Accurate categorization of pregnancies (IVF, IVF + PGT-A, or natural conception) was confirmed by a chart review. Fetal anomaly data was obtained from the targeted anatomy ultrasound report and categorized as major or minor and by organ system according to the Center for Disease Control and Prevention (CDC) guidelines [16]. Abnormal placental findings noted on anatomy ultrasound including placenta previa, velamentous cord insertion, single umbilical artery, presence of a succenturiate placental lobe, and evidence of placenta accreta were also recorded. Patient demographics, including body mass index (BMI), parity, and maternal comorbidities were extracted. Maternal comorbidities reviewed included hypertensive disorders, gestational and pregestational diabetes, autoimmune disease, and cardiac disorders. The primary outcome was the rate of anomalies detected at the time of the anatomy ultrasound. Secondary outcomes included rate of abnormal nuchal translucency (NT), abnormal second trimester serum analytes, high-risk non-invasive prenatal testing (NIPT), and abnormal invasive diagnostic testing (chorionic villus sampling (CVS) and amniocentesis). NT measurements were performed by sonographers and physicians certified by the NT Quality Review Program. NT was classified as abnormal if the measurement was greater than or equal to 3 mm [17]. Second trimester serum screen consisted of human chorionic gonadotropin, alpha fetoprotein, inhibin A, and unconjugated estriol [18, 19]. Abnormal second trimester screen was determined by the California Prenatal Screening Program age-based cutoffs for serum analytes. Patients who underwent diagnostic testing via CVS or amniocentesis had genetic testing through different platforms based on indication for testing. The platforms used included karyotype, chromosomal microarray (CMA), and fluorescent in situ hybridization (FISH). Indications for invasive diagnostic testing included abnormal ultrasound finding, genetic abnormality in a prior pregnancy, personal or family history of genetic disorder, and advanced maternal age.

### Statistical analysis

To detect a reduction in ultrasound-detected anomalies from 3 to 1% (OR 0.33), 768 patients would be needed per group. Our convenience sample of 2 years of ultrasounds performed on patients who had undergone IVF yielded 712 patients. A power calculation indicated that a sample size of 710 patients per group would afford 80% power to detect an OR of 0.27, or a difference from a baseline rate of ultrasound-detected anomalies of 3 to 0.8%.

Comparison of continuous variables was performed using Student’s *t* test. Categorical variables were compared using the chi-square or fisher’s exact test where applicable. Logistic regression was used for multivariate analysis. Statistical significance was set at a *p* value of < 0.05 with two-tailed testing. Statistical analyses were performed using STATA version 14 (Stata Corp., College Station, TX).

### Ethical approval

This study was approved by the Institutional Review Board of the University of California, Los Angeles (IRB# 19-000222).

## Results

A total of 712 patients with singleton pregnancies who conceived with IVF had a mid-trimester ultrasound performed during the study period. Compared to age-matched natural conception controls, patients who conceived after IVF had significantly lower parity (0.6 ± 0.9 vs 0.9 ± 1.1, *p* < 0.001) and a higher rate of maternal comorbidities (17.3% vs 11.7%, *p* = 0.002). There were no differences in BMI (Table 1). Of the IVF conception patients, 236 (33%) conceived after IVF with unscreened embryos and 476 (67%) conceived after IVF with PGT-A. Among autologous IVF cycles, there was no difference in patient age between those who underwent PGT-A and those who did not. Patients who conceived after IVF with unscreened embryos were more likely to have conceived after oocyte donation (28.4% vs 13.9%, *p* < 0.001). The average age of the egg donor was also younger in IVF with the unscreened embryo group (26.7 ± 4.8 years vs 28.6 ± 5.7 years, *p* = 0.038). BMI, parity, rate of comorbidities, and use of gestational carrier did not differ between the two groups (Table 2).

**Table 1:** Patient demographics—natural conception vs IVF

	Natural conception (*n* = 712)	IVF (*n* = 712)	*p* value
Age	35.0 ± 5.4	35.0 ± 5.4	1^a^
Parity	0.9 ± 1.1	0.6 ± 0.9	< 0.001^a^
BMI	24.1 ± 4.4	24.3 ± 3.9	0.515^a^
Comorbidities^c^	83 (11.7%)	123 (17.3%)	0.002^b^

Data presented as mean ± standard deviation for parametric continuous variables and *n* (%) for categorical variables

^a^*t* test

^b^Chi-square test

^c^Characterization of comorbidities is detailed in Supplemental Table 1

**Table 2:** Patient demographics—IVF no PGT vs IVF + PGT

	IVF no PGT (*n* = 237)	IVF + PGT (*n* = 475)	*p* value
Age (autologous cycles)	36.5 ± 3.9	36.7 ± 3.8	0.696^a^
Age (oocyte donation cycles)	26.7 ± 4.8 (*n* = 67)	28.6 ± 5.7 (*n* = 66)	0.038^a^
Parity	0.6 ± 0.8	0.6 ± 0.9	0.672^b^
BMI	24.7 ± 4.3	24.1 ± 3.8	0.053^a^
Comorbidities	45 (19.0%)	78 (16.4%)	0.380^b^
Gestational carrier	14 (5.9%)	25 (5.3%)	0.707^b^

Data presented as mean ± standard deviation for parametric continuous variables and *n* (%) for categorical variables

^a^*t* test

^b^Chi-square test

There were no significant differences in the rate of abnormal anatomy ultrasound, detection of major or minor fetal anomalies, or rate of high-risk NT in patients who underwent IVF with PGT-A compared to patients who conceived following IVF with unscreened embryos and age-matched patients with natural conceptions. The characterization of major and minor anomalies by group is shown in Table 4. Several fetuses with anomalous findings on ultrasound had abnormalities of multiple systems. Table 3 presents the rates of anomalous fetuses per group on a per-patient basis, while Table 4 displays the total number of major and minor anomalies per group. Patients with natural conceptions had a significantly lower rate of abnormal placental findings compared to patients who conceived after IVF with unscreened embryos or IVF with PGT-A (2.9% vs 8.9% vs 8.8%, *p* < 0.001) (Table 3). This finding of an increased rate of placental abnormalities in patients who conceived after IVF with or without PGT-A when compared to natural conceptions persisted in a multivariate logistic regression model adjusting for the presence of medical comorbidities in the mother (*p* < 0.001). The breakdown in frequency of various placental anomalies is available in Supplemental Table 2. There was no difference in rate of placental anomalies between oocyte donor and autologous IVF pregnancies.

**Table 3:** Sonographic evaluation

	Natural conception	IVF no PGT	IVF + PGT	*p* value
High-risk NT	2/513 (0.4%)	2/175 (1.1%)	3/371 (0.8%)	0.350^b^
Anatomy US abnormal	77/712 (10.8%)	36/236 (15.2%)	68/475 (14.3%)	0.093^a^
Placenta US abnormal	21/712 (3.0%)	21/236 (8.9%)	42/475 (8.8%)	< 0.001^a^

Data presented *n* (%), with denominators provided when data is missing

^a^Chi-square test

^b^Fisher’s exact test

**Table 4:** Fetal anomaly characterization

	Natural conception	IVF no PGT	IVF + PGT	*p* value
Major anomalies	27 (3.8%)	10 (4.2%)	18 (3.8%)	0.948^a^
Nervous	5	0	2	
Cardiac	6	7	8	
GI	1	0	1	
GU	5	1	1	
MSK/bone	4	1	3	
Cleft lip/palate	1	0	0	
Metabolic	0	0	0	
Malformation syndrome	1	1	2	
Other	4	0	2	
Minor anomalies	72 (10.1%)	28 (11.9%)	58 (12.2%)	0.494^a^
Nervous	13	1	3	
Cardiac	31	17	24	
GI	12	3	7	
GU	20	6	24	
Other	6	4	11	

Data presented *n* (%)

*GI* gastrointestinal, *GU* genitourinary, *MSK* musculoskeletal

^a^Chi-square test

The rate of high-risk NIPT did not differ among patients who underwent IVF with PGT-A compared to patients who conceived following IVF with unscreened embryos and age-matched patients with natural conceptions. Compared to patients who conceived after IVF with unscreened embryos and IVF with PGT-A, patients with natural conceptions had a lower rate of abnormal second trimester serum analytes (0.9% vs 3.5% vs 4.5%, *p* = 0.003). This finding of a difference in rate of abnormal second trimester screen between treatment groups persisted in a multivariate logistic regression adjusting for the presence of medical comorbidities in the mother (*p* = 0.002). A total of 131 patients elected to undergo invasive diagnostic testing via amniocentesis/CVS. The rate of abnormal invasive diagnostic testing was significantly higher in patients with natural conceptions compared to patients who conceived after IVF with unscreened embryos or IVF with PGT-A (12.0% vs 5.9% vs 0%, *p* = 0.049) (Table 5). Genetic abnormalities included segmental and whole chromosome mosaicism, microdeletions or microduplications, whole chromosome aneuploidy, and 47 XXX. Notably, there was 100% concordance of PGT-A with normal karyotype on invasive diagnostic testing though the number of patients who pursued invasive testing was low overall.

**Table 5:** Serum screening and invasive diagnostic testing

	Natural conception	IVF no PGT	IVF + PGT	*p* value^a^
NIPT high-risk	9/622 (1.5%)	1/186 (0.5%)	3/395 (0.8%)	0.550
Second trimester serum analyte abnormal	5/537 (0.9%)	6/172 (3.5%)	16/358 (4.5%)	0.002
Invasive diagnostic testing abnormal	9/75 (12.0%)	1/17 (5.9%)	0/39 (0%)	0.049

Data presented as *n* (%), with denominators provided when data is missing

^a^Fisher’s exact test

## Discussion

In this large cohort, the rate of fetal anomalies detected on mid-trimester ultrasound did not differ in patients who conceived after IVF with PGT-A compared to patients who conceived following IVF with unscreened embryos and age-matched patients with natural conceptions. Rates of abnormal NT and NIPT were also similar between groups. These findings are consistent with prior studies that have reported similar rates of congenital anomalies in neonates conceived through IVF with PGT compared to those conceived with unscreened embryos [15, 20–23]. However, nearly all prior studies involved cleavage stage or polar body biopsy for PGD with FISH or array comparative genome hybridization. While we do not have data on the biopsy technique or testing platform, our cohort was derived from 2017 to 2018, at which time blastocyst stage trophectoderm biopsy with next-generation sequencing was overwhelmingly adopted [6, 24, 25].

A significantly higher rate of abnormal placental ultrasound findings and abnormal second trimester serum analytes were seen in patients who conceived following IVF with or without PGT-A compared to natural conception controls. Placental complications such as placental insufficiency and preeclampsia have been associated with alterations in second trimester maternal serum analytes, and patients with subfertility who conceive with and without IVF have been demonstrated to have higher rates of abnormal placentation in several large studies [26–30]. The increased rates of abnormal second trimester serum analytes and abnormal placental appearance on mid-trimester ultrasound among patients who conceived after IVF in our study are therefore consistent with prior research. The frequency of detection of subgroups of placental abnormalities was small and, therefore, meaningful comparisons could not be made for specific groups of placental abnormalities. This should be investigated further. We did not, however, see a difference in placental abnormalities nor abnormal serum analytes between IVF and IVF with PGT-A. This is consistent with a recent retrospective cohort study which found no difference in rate of abnormal placentation in patients who conceived after IVF with or without PGT-A [31].

The rate of abnormal invasive diagnostic testing was significantly higher among patients with natural conceptions compared to patients who conceived following IVF with or without PGT-A, with a *p* value that just met significance, *p* = 0.049. This difference was driven by a 0% abnormal invasive testing rate in the IVF with the PGT-A group, who accounted 39 of the 56 IVF patients who underwent invasive testing. This result was not surprising given that PGT-A would be expected to reduce the number of chromosomal abnormalities detectable on CVS/amniocentesis karyotype. While discordance between PGT-A and invasive diagnostic testing may occur in rare cases, we would not expect to see it in our cohort given the sample size.

Previous studies have shown that patients have misconceptions about the utility of PGT-A and that this impacts decision-making regarding prenatal care. In a prior publication by one of the authors, the majority of patients undergoing IVF believed PGT-A would improve the chance of having a “healthy baby” and reduce the risk of birth defects [7]. A study evaluating the utilization of prenatal genetic screening among patients at an academic reproductive endocrinology and infertility (REI) clinic found that patients who conceived after IVF with PGT-A were more likely to decline all prenatal screening and diagnostic testing than patients who conceived with unscreened embryos, 19.1% vs 4% respectively [32]. The results of the present study provide evidence that PGT-A does not preclude or even significantly reduce the risk of fetal anomalies in pregnancies conceived with IVF. This highlights the importance of improved patient counseling regarding the limitations of PGT-A, and of providing standard prenatal care for pregnancies conceived through ART, including prenatal aneuploidy screening and diagnostic testing, regardless of whether PGT was performed.

Limitations of this study include its retrospective design and the potential for selection bias given that the study setting is a high-risk referral center. The rates of anomalies in this patient population, therefore, may not reflect those in the general population. We aimed to mitigate this by inclusion of a natural conception control group in which we found a baseline incidence of anomalies similar to what has previously reported in the general population. We did not include birth outcome, newborn exam data, or placental pathology data and therefore could not corroborate the anomalies detected on mid-trimester ultrasound with clinical outcomes. While this is a limitation, it is worth noting that many pregnancies complicated by major anomalies detected on mid-trimester ultrasound are terminated and, thus, would not necessarily be captured by inclusion of newborn examination data. Finally, because only women who underwent mid-trimester ultrasound were included, this would not capture early miscarriages or terminations.

Strengths of our study include the large sample size, inclusion of a natural conception control group, and that all analyzed data originated from 2017 to 2018, which increases the likelihood that IVF and PGT-A technologies used were more homogenous. Additionally, to our knowledge, this is the first study to report the rate of ultrasound anomalies, fetal and placental, in IVF patients with and without PGT-A.

In conclusion, the use of PGT-A was not associated with a difference in the rate of fetal anomalies detected on mid-trimester ultrasound in this large cohort. While the available evidence does not support the use of PGT-A for the purpose of reducing the risk of birth defects, patients consistently report this as a primary reason for pursuing this technology [7]. Improvement in patient counseling regarding the limitations of PGT-A is crucial.

## Data Availability

Coded data pertaining to this study are stored on a password and firewall protected server and can be made available if needed.

## Supplemental information

ESM 1 (DOCX 12 kb).
