# Supplementary material for: Sonographic abnormalities in pregnancies conceived following IVF with and without preimplantation genetic testing for aneuploidy (PGT-A)
Source: J Assist Reprod Genet. 2021 Feb 3;38(4):865–71. doi: 10.1007/s10815-021-02069-5 (PMC8079593; doi:10.1007/s10815-021-02069-5)
Supplement: ESM 1 — (DOCX 12 kb). [file 10815_2021_2069_MOESM1_ESM.docx]

**Supplemental Table 1.** **Characterization of comorbidities – Natural conception vs IVF**

|  | | Natural conception (n=712) | IVF  (n=712) |
| --- | --- | --- | --- |
| Comorbidity | | 83/712 (11.7%) | 123/712 (17.3%) |
|  | Alcohol exposure | 3/3 | 0/3 |
|  | Autoimmune disease | 4/17 | 13/17 |
|  | Cardiac disorders | 0/5 | 5/5 |
|  | Gestational diabetes | 45/93 | 48/93 |
|  | History of malignancy | 1/3 | 2/3 |
|  | Hypertensive disorders | 11/38 | 27/38 |
|  | Pregestational diabetes | 8/13 | 5/13 |
|  | Psychiatric disorders | 7/18 | 11/18 |
|  | Smoker | 1/1 | 0/1 |
|  | Thyroid disease | 11/48 | 37/48 |
|  | Uterine malformations | 1/2 | 1/2 |
|  | Other | 5/11 | 6/11 |

**Supplemental Table 2. Characterization of placental abnormalities – Natural conception vs IVF no PGT vs IVF+PGT**

|  | Natural conception  (n=712) | IVF no PGT  (n=237) | IVF+PGT  (n=475) |
| --- | --- | --- | --- |
| Placenta previa | 7 (1.0) | 4 (1.7) | 15 (3.2) |
| SUA | 9 (1.3) | 4 (1.7) | 6 (1.3) |
| Velamentous cord insertion | 4 (0.6) | 11 (4.6) | 11 (2.3) |
| Succenturiate lobe | 0 (0.0) | 1 (0.4) | 1 (0.2) |
| Placenta accreta | 2 (0.3) | 2 (0.8) | 10 (2.1) |

Data presented as n (%).

SUA – single umbilical artery
